# Continuity of care in crisis: Community based mobile health teams for sexual and reproductive health services in post-earthquake period in Türkiye

**DOI:** 10.3389/fpubh.2025.1706038

**Published:** 2026-01-15

**Authors:** Derya Çamur, Ahmet Can Bilgin, Tacettin Inandi, Nazan Savas, Öykü Turunç, Bengü Nehir Bugdayci Yalçin, Zehra Kilinc, Bulent Kilic

**Affiliations:** 1 Department of Public Health, Gülhane Faculty of Medicine, University of Health Sciences, Ankara, Türkiye; 2 Department of History of Medicine and Ethics, Faculty of Medicine, Dokuz Eylül University, Izmir, Türkiye; 3 Department of Public Health, Tayfur Ata Sökmen Faculty of Medicine, Hatay Mustafa Kemal University, Hatay, Türkiye; 4 Department of Public Health, Faculty of Medicine, Koç University, Istanbul, Türkiye; 5 Department of Public Health, Faculty of Medicine, Mersin University, Mersin, Türkiye; 6 Department of Public Health, Faculty of Medicine, Dicle University, Diyarbakir, Türkiye; 7 Department of Public Health, Faculty of Medicine, Dokuz Eylül University, Izmir, Türkiye

**Keywords:** inter-sectoral collaboration, mobile services, NGOs, region-based health, social determinants

## Abstract

**Introduction:**

More than 50 thousand people lost their lives in the earthquake that occurred in Türkiye in 2023. This study aimed to evaluate the Sexual and Reproductive Health (SRH) services provided in earthquake region in Hatay province in Türkiye with qualitative and quantitative methods.

**Materials and methods:**

The research type is mixed; cross-sectional and phenomenological. The recorded data were evaluated for cross-sectional phase and purposeful and snowball sampling was conducted for qualitative phase. Data were collected through five focus group interviews (one healthcare workers group-−5 staff- and four women group-−16 women-) and 13 in-depth interviews (4 academics, 6 NGO, 3 service providers) as totally 34 participants. Thematic content analysis was conducted.

**Results:**

Eighty three percentage of 15,841 women's applications were reached by mobile services. All of women were given health education. The frequency of genitourinary infection is 25.0%. Family Planning (FP) method was given to 35.3% of the women, cotton underwear was given to 72.0%, and sanitary pads were given to 22%. Emerging themes included social determinants, the gap in SRH services, community based mobile services and service provision by NGOs like HASUDER (Association of Public Health Specialists) and intersectoral collaboration. Reported that pre-existing gaps in SRH services were exacerbated after the earthquake, with major barriers including lack of privacy, poor hygiene, limited family planning access, low SRH awareness. Related to social determinants, gender and migration-related challenges such as language barriers, economic hardship, cultural isolation restricted service access. NGOs played a crucial role in addressing unmet needs. However, bureaucratic barriers and limited public sector collaboration were noted. Deteriorated living conditions, and widespread healthcare disruptions across all service levels further deepened vulnerabilities. The HASUDER mobile services were highlighted as critical in reaching women, adolescents, and migrants.

**Discussion:**

The earthquake deepened health system gaps, while NGOs became essential in bridging services and building resilience. Strengthened coordination, infrastructure, and inclusive SRH integration are urgently needed. Community-based mobile service delivery is vital.

## 1 Introduction

On February 6, 2023, two earthquakes with magnitudes of 7.7 Mw and 7.6 Mw, both with epicenters in the province of Kahramanmaraş, struck nine hours apart, which came to be known as the 2023 Türkiye–Syria earthquakes (1, 2). The earthquakes affected 11 provinces, caused widespread destruction, and resulted in 53,537 officially reported deaths (3). Hatay was the most severely affected province, with approximately 24,000 fatalities (4). In Hatay, the earthquakes had a profound impact on health service delivery: hospital and primary healthcare facilities collapsed, a substantial number of healthcare workers lost their lives, and service provision was brought to a near standstill.

The Association of Public Health Specialists (HASUDER) is a professional organization composed of physicians specialized in public health in Türkiye (5). As a non-governmental organization (NGO), HASUDER initiated a project on sexual and reproductive health (SRH) services in Hatay, the province most severely affected, and began service provision on March 15, 2023. Throughout the project, all services and supplies, including pharmaceuticals, contraceptives, hygiene kits, and essential clothing items, were provided free of charge to beneficiaries.

Continuity of care in crisis is a very important problem. The main problem is the unmet need for services. The increase in sexual and reproductive health (SRH) needs during crises and disasters, combined with the tendency for SRH service provision to remain neglected in the face of these growing needs, constituted the key determinants for selecting SRH as the focus of the project and service delivery.

In humanitarian crises, SRH services are frequently deprioritized, creating an unmet gap in service provision (6–14). Factors such as the narrowing or loss of spaces ensuring privacy, difficulties in accessing healthcare, and the reduced capacity of health facilities further heighten the importance of SRH services (10, 15, 16). The risk of sexually transmitted infections (STIs), including HIV, unsafe abortions and deliveries, cases of gender-based violence, and the heightened vulnerability of women and girls all contribute to the increased demand for SRH services (17). Interruptions in SRH service delivery may lead to higher maternal and child mortality, unintended pregnancies, unsafe (induced) abortions, or unwanted births (18, 19). Therefore, the planning, coordination, provision, and accessibility of SRH services during disasters are of critical importance (20).

An important conceptual linkage can be identified between the gap in SRH services and the framework of the Social Determinants of Health. Structural factors such as poverty, unemployment, cultural norms, gender dynamics, and housing conditions exert a profound influence on the accessibility and utilization of SRH services. These determinants collectively underscore the critical need to situate SRH inequities within broader socio-economic and environmental contexts. The exacerbation of such disparities during crisis situations further illustrates the interdependence between social vulnerability and health outcomes. Consequently, addressing the SRH service gap requires a multidimensional approach that integrates community-based mobile health initiatives and fosters inter-sectoral collaboration. Such strategies are essential for mitigating structural inequities and promoting sustainable, equitable access to SRH care. Therefore, this study focuses on how SRH services can be improved and how access to services can be increased in the post-earthquake emergency environment.

To ensure SRH service delivery in humanitarian crises, the United Nations Inter-Agency Working Group (IAWG) developed the Minimum Initial Service Package for Sexual and Reproductive Health in Crisis Situations (MISP), which emphasizes the integration of MISP into every humanitarian intervention from the earliest days of response through the recovery phase (20, 21). Furthermore, the recent discontinuation of publicly provided free family planning services in Türkiye, together with the increase in unmet need for family planning from 6% in 2013 to 12% in 2018 (22), underscores that reproductive health and family planning services will remain a priority in the post-disaster period.

Anticipating the need for services in the field SRH during the acute phase following the earthquake, HASUDER developed a field implementation project and mobilized financial resources through international collaborations with the International Planned Parenthood Federation-IPPF (March 15–June 15, 2023), Direct Relief-DR (June 15, 2023–February 28, 2025), and United Nations Population Fund-UNFPA (August 1, 2023–February 28, 2025). All units provided community-based SRH services through fixed and mobile delivery models. To ensure local coordination, official approval was obtained from the Governor's Office, and cooperation protocols were signed with the Hatay Metropolitan Municipality and the Hatay Provincial Health Directorate.

Nevertheless, women's access to the unit was limited due to acute-phase circumstances, including widespread destruction, the absence of public transportation, damage to private vehicles, the loss of family members, lack of childcare, and economic hardship. These constraints underscored the need for mobile service delivery to ensure broader access. Due to the considerable service gap two panel van was supplied for mobile service provision, and one physician, four nurses/midwives, and two drivers were employed. Since the services were directed toward women's SRH needs, all staff members except the drivers were women, a deliberate choice to enhance the acceptability of services. The service model was predominantly transformed into a mobile format, with the vehicles internally designed to accommodate clinical consultations. These two units were strategically located in different parts of the city, with defined catchment areas. The primary target population consisted of women of reproductive age (15–49 years), although older women who sought care were not turned away. In this way, community-based service delivery was organized through two separate teams. In Hatay, where the Syrian migrant population is substantial, services were delivered irrespective of ethnic background. As all staff were fluent in Turkish and Arabic, language barriers were not encountered. Furthermore, personnel participated in and completed both in-person and online SRH trainings organized by the Ministry of Health, UNFPA, and HASUDER.

Mobile visits were conducted for two purposes: the first involved short introductory visits for outreach and service planning, and the second were service visits. During introductory visits, the teams typically contacted the managers of temporary settlements, family physicians, local headmen (muhtars), or community leaders referred to as “female community leaders” to present the scope of services. If accepted, a plan was made and followed by a second visit for service delivery. During service visits, educational sessions were conducted with groups of 30–40 women, followed by individual consultations. When necessary, women received medical treatment, family planning services and supplies, and hygiene materials. All activities of the unit were documented through daily physician reports and individual forms. Each team was provided with essential equipment, including medications, pregnancy tests, family planning supplies, and educational tools.

Throughout the entire service period, sustained collaboration was ensured with District Health Directorates, the university hospital, the state hospital, the Disaster and Emergency Management Authority under the Ministry of Interior (AFAD), the Hatay Medical Chamber, as well as with the managers and health units of temporary settlements. Public health academics who were members of HASUDER provided expert consultation to the teams.

HASUDER's mobile health intervention were also consistent with other international standards. When benchmarked against the Sphere Project Standards (23) and WHO guidelines on SRH in emergencies (24), HASUDER's approach also reflected adherence to key principles such as accessibility, quality of care, and accountability. The use of mobile health teams, provision of family planning services, treatment of infections, and distribution of hygiene materials correspond closely to the Sphere health standards concerning essential healthcare and dignity in service delivery. However, areas of partial divergence were identified, particularly in the absence of a formal monitoring and evaluation framework to systematically assess service quality and outcomes, as recommended by WHO and Sphere. Overall, the intervention can be regarded as largely consistent with international humanitarian health standards, while highlighting the need for enhanced documentation and continuous quality assurance mechanisms to strengthen future implementations.

This study was conducted to evaluate the SRH services provided in Hatay by HASUDER after the February 6, 2023 earthquakes through a multisectoral, community-based, and predominantly mobile service model, using both quantitative and qualitative methods from the perspectives of service recipients and providers.

## 2 Materials and methods

This research employed a mixed-methods design, combining a quantitative descriptive cross-sectional study with a qualitative phenomenological approach. The mixed method was chosen because of the inadequacy of data obtained using quantitative methods. Assessments based solely on records cannot understand women's expectations and the experiences of service providers. Due to the lack of sufficient data, particularly on intersectoral collaboration and social determinants, the study design was supported by a phenomenological approach.

Each method was designed to address the research objectives. Quantitative methods aimed to determine how many women applied, what their reasons were, and service trends. Qualitative methods aimed to identify the expectations, experiences, and solution recommendations of women and healthcare providers.

The study primarily analyzed quantitative data, and for issues that the available data could not identify, it was decided to proceed with a qualitative approach. This aimed to identify the causes and solutions to women's problems in the SRH area. When analyzing qualitative data, care was taken to integrate it with the available quantitative data.

Ethical approval was obtained from the Hatay Mustafa Kemal University Non-Interventional Clinical and Research Ethics Committee (approval dated January 8, 2025, No. 63). In addition, permissions for data use were granted by UNFPA, Direct Relief, and HASUDER, and voluntary informed consent was obtained from all participants.

### 2.1 Quantitative component (descriptive cross-sectional)

This component analyzed the records of women who received services from the two HASUDER Women's and Reproductive Health Units in Hatay between June 15, 2023, and February 28, 2025. Variables assessed included age, nationality, service delivery modality (fixed unit or mobile), and the type of SRH services provided. Data from the initial three-month period (March 15–June 15, 2023) were excluded due to incomplete documentation during the acute post-disaster phase. Descriptive statistics, including frequencies and percentages, were used to summarize the data.

### 2.2 Qualitative component

In the qualitative component, a phenomenological design was applied to explore how social phenomena are situated in the everyday lives of actors, based on their lived experiences in the post-disaster period. The phenomenological framework guided both the development of research questions and the analytical process by centring the lived experiences of women affected by the earthquake. This approach prioritized understanding how participants perceived and made sense of the challenges surrounding access to SRH services within their specific social and cultural contexts. Consequently, the research questions were designed to elicit detailed narratives about personal experiences, barriers, and coping mechanisms rather than to quantify service use. During analysis, phenomenology informed the thematic interpretation by focusing on the essence of participants' experiences shaped their access to SRH services. This framework also allowed the researcher to identify underlying meanings behind these experiences, such as feelings of exclusion, vulnerability, and resilience. Thus, the phenomenological lens ensured that the study captured not only the structural determinants of SRH gaps but also the subjective realities of women navigating these crises.

Qualitative data were collected face-to-face by the research team in Hatay. Purposive and snowball sampling methods were chosen to reach disadvantaged women and key informants in the region. In the first stage of sample selection, contact was made with NGOs operating in the earthquake zone, and six NGOs with the longest service delivery in the region were identified. Then, interviews were conducted with representatives of these NGOs to request their assistance in reaching women living in temporary settlements. In this way, volunteer women were found in two separate temporary settlement areas, and the women to be interviewed in the focus group were reached. This allowed us to reach Syrian refugee women and women from groups with limited access to services. Additionally, in line with the research aim, data was collected from academics and doctors from the Obstetrics and Gynecology and Public Health departments of university and state hospital in the region who agreed to participate in the interviews. There were only one university and one state hospital in the region. Additionally, at least one employee from each of the local NGOs was selected for in-depth interviews.

Data collection was concluded once saturation was reached. Three separate teams, comprising eight researchers, were formed to conduct interviews during the data collection phase of the study. At the end of each day, the teams met to discuss the interviews and data. Once all interviews were completed, the researchers reviewed the data, transcriptions and reached a consensus decision that data saturation had been achieved. Data saturation was also evaluated after the analyses were completed.

Data were gathered in January–February 2025 through 5 focus group discussions involving health workers (one group, 5 people), Turkish women (3 groups, 12 people), and Syrian migrant women (one group, 4 people). In addition, data were collected through 13 in-depth interviews (4 academics, 6 NGO, 3 service providers). Totally 34 participants were interviewed in the research. The discussions explored comparisons of SRH services before and after the earthquake, perceived needs and service gaps, perspectives on a multisectoral model of SRH service provision, and participants' feelings, perceptions, and suggestions regarding SRH services. Semi-structured interview guides were used.

A total of five focus group discussions were conducted, with an average duration of 77 min, and 13 in-depth interviews (seven with women and six with men) with an average duration of 52 min. All interviews were conducted face-to-face with participants' written and verbal consent and were audio-recorded. Each interview was facilitated by two researchers, one of whom acted as rapporteur. For migrant women or those not fluent in the language, a female translator was present.

Interview guides were developed by the researchers drawing on relevant literature and their own professional experience. The questions were designed to allow participants to express their own views and perspectives. At the end of each interview, audio recordings were transcribed into written texts, which were returned to participants for confirmation. The transcripts were then independently coded manually by two different researchers. Codes were consolidated in collaboration with the research team, and a thematic analysis was conducted following consensus on the coding framework. All interviews were coded by at least two different researchers. After all coding was completed, DÇ, ACB, and BK combined these codes to achieve intercoder reliability. Finally, the entire team finalized the codes and themes. The researchers did not use any qualitative software, all the analyses are realized by manually.

To assess the *credibility* criterion of the study, the findings derived from the analysis were shared with a subset of participants to obtain their feedback for participant validation. Triangulation was also carried out from the three different data sources (service providers, service users and NGOs). The fact that two researchers (TI and NS) worked in the study area and were familiar with the local context was considered to enhance the credibility of the research. To ensure *transferability*, detailed information on the characteristics of the participants and the study setting was provided, allowing other researchers to obtain comparable results in similar contexts with similar participants. In line with the *confirmability* criterion, direct quotations from participants' own statements as descriptive evidence were included in the presentation of the findings prior to interpretation. For *dependability*, the coding process was independently conducted by two researchers.

## 3 Results

The findings revealed compelling themes that align closely with the study's objectives, demonstrating a strong interconnection between the challenges experienced by women and the availability of SRH services. The overarching theme emerging from the analysis was the influence of social determinants on SRH outcomes. It was observed that women's limited access to family planning counseling was shaped by intersecting factors such as gender inequality, migration, poverty, and low levels of education. In addition, the high prevalence of sexually transmitted infections underscored the broader systemic gaps in SRH service provision. The findings further emphasized the critical importance of maintaining service continuity during crisis situations. Accordingly, crisis preparedness, effective coordination mechanisms, and intersectoral collaboration should be prioritized as essential strategies to ensure equitable and sustained access to SRH services.

### 3.1 Quantitative findings

Between June 15, 2023, and February 28, 2025, a total of 15,841 service contacts with women were recorded at the two HASUDER Women's and Reproductive Health Units. The mean age of the women was 38.0 ± 12.9 years; 80.2% were Turkish citizens and 14.7% were Syrian migrants. The majority of women (83.0%) were reached through mobile services. All women received health education on topics such as personal hygiene, handwashing, sexually transmitted infections, breast self-examination, breastfeeding, and Kegel exercises.

Among the health services provided, the treatment of sexually transmitted infections (STIs) and urinary tract infections (25.0%) ranked first. Women diagnosed with STIs and urinary tract infections received pharmacological treatment, while 3.9% were referred to the gynecology and obstetrics clinics of the Faculty of Medicine or the state hospital for further evaluation and treatment. The most common genitourinary symptom was vaginal discharge (18.9%). A total of 818 antenatal follow-ups were conducted. Within the scope of family planning services, following general and method-specific counseling, the most commonly distributed commodities were male condoms (24.5%) and oral contraceptives (7.3%). In terms of hygiene supplies, 72.0% of women received underwear and 22.0% received sanitary pads (Table 1).

**Table 1:** Community-based and mobile sexual and reproductive health service interventions implemented by HASUDER in Hatay following the earthquake (15 June 2023–28 February 2025; *n*: 15,841).

**Characteristics**	**Number**	**Percent^a^**
**Age**	Mean ± SD = 38.0 ± 12.9, median = 36.0	
	Min–max = 15–98	
**Nationality**		
Turkish citizens	12,224	80.2
Syrian migrants	2,246	14.7
Migrants from another countries	775	5.1
**Service delivery setting**		
Mobile services	13,152	83.0
Fixed units	2,689	17.0
**Women receiving health education**	15,841	100.0
**Women referred to hospital**	633	3.9
**Health services provided**		
Treatment of sexually transmitted infections	2,997	18.9
Treatment of urinary tract infections	980	6.1
Antenatal care	818	5.2
Pregnancy test	264	1.7
**Family planning commodities provided**		
Male condom	3,884	24.5
Oral contraceptive	1,152	7.3
IUD	404	2.6
Injectable contraceptive	109	0.7
Emergency contraceptive pill	23	0.2
**Hygiene supplies provided**		
Cotton underwear	11,410	72.0
Menstrual pad	3,485	22.0
Emergency birth kit	738	4.7
Dignity kit	574	3.6

^a^Percentages were calculated based on different denominators: age (*n* = 12,317), nationality (*n* = 15,245), and all other variables (*n* = 15,841).

### 3.2 Qualitative findings

**Figure 1:**
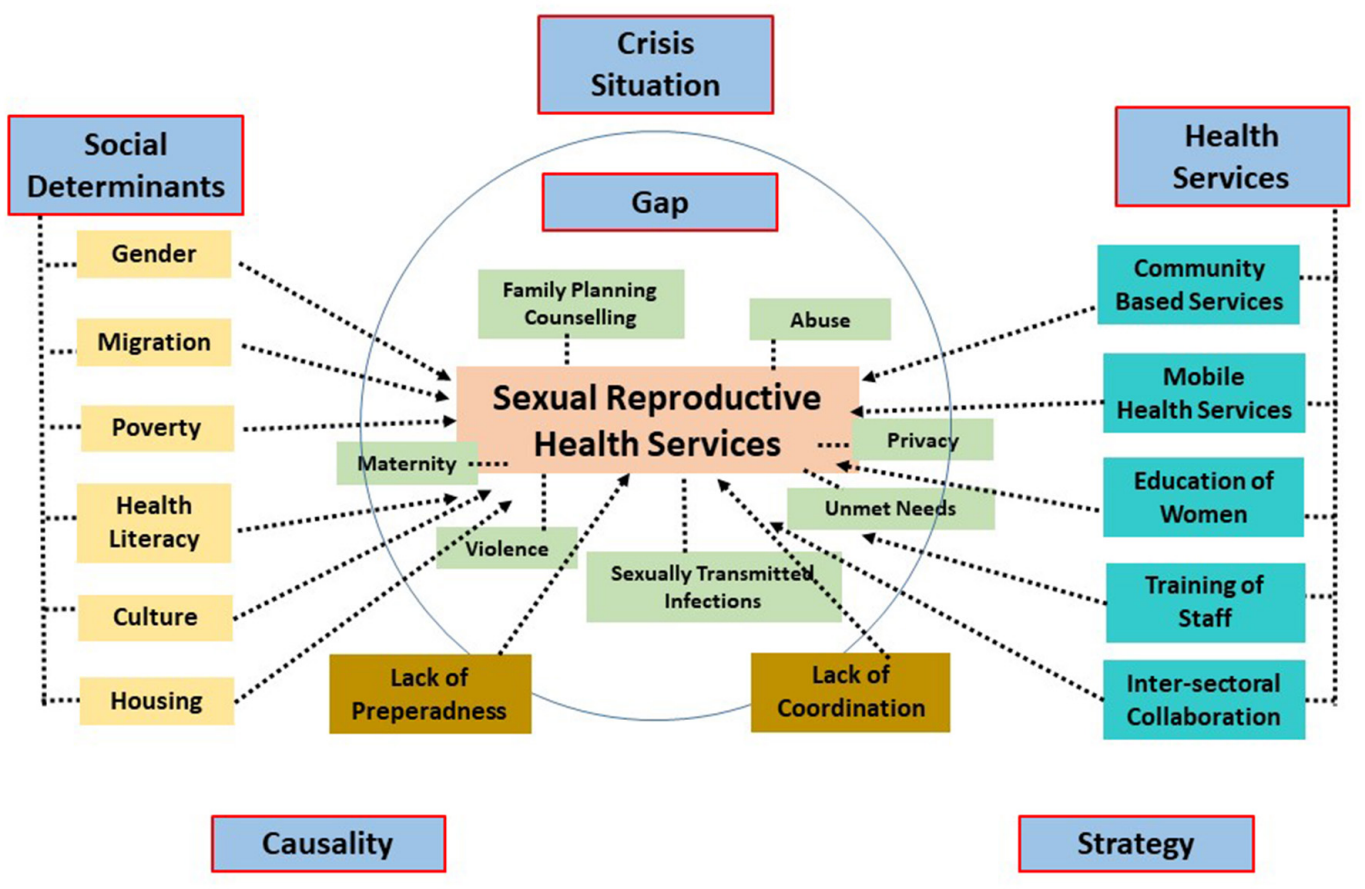
Coding tree and conceptual framework illustrating the provision and determinants of sexual and reproductive health services in Hatay following the 2023 earthquakes.

At the end of this study, four major themes emerged: *The Gap in SRH Services, Social Determinants of Health, Community-based Mobile Services, and Inter-sectoral Collaboration*. These themes highlight the increasing importance of SRH services in crisis situations and demonstrate that the gap in SRH is closely linked to the social determinants of health. Furthermore, they indicate that community-based mobile services and inter-sectoral collaboration should be regarded as essential components in addressing these challenges. This conceptual framework, along with the coding tree of the study, is presented in Figure 1.

#### 3.2.1 The gap in SRH services

The importance of sexual and reproductive health (SRH) services in disaster settings was initially underrecognized even among healthcare professionals; however, the significance of this area became evident once service delivery was initiated.

“*At first, we couldn't really connect sexual health with disasters. Even though we were healthcare workers, it felt strange. But once we got involved, we realized how valuable and important it actually was.”*

*EG, 28 years, Female, Physician*

“*In the acute phase, these kinds of reproductive health services were really left in the background. Even health professionals didn't see them as an urgent need. Because of this bias, accessibility was also affected.”*

*AU, 30 years, Male, Physician*

The gap in SRH services further deepened in the aftermath of the earthquake. One in four women in the region reported symptoms of sexually transmitted infections. Vaginal discharge was identified as the most common sexual health problem, accounting for 18.9% of cases, followed by urinary tract infections at 6.2%.

“*…She was pregnant… when you're pregnant you get itching. There was burning when urinating as well. She would always come here.”*

*SA, 19 years, Female, One child, Housewife*

“*…When my period was delayed, I came here for a pregnancy test. I also benefited from birth control pills. Other than that, they gave me medicine here for odor and discharge.”*

*SPK, 26 years, Female, One child, Housewife*

The second most common reason for women's visits was family planning counseling. In this project, 35.3% of women who sought services received contraceptive commodities. However, the persistence of unprotected sexual intercourse has led to unintended pregnancies, underscoring the unmet need in this area.

“*I was going to get birth control pills. I asked at the pharmacy. They told me 500 TL (13 USD) just for one pack. Then I heard about this place. Here they gave it to us for free.”*

*ZA, 18 years, Female, No children, Housewife*

“*There was a lady with five children. She came here to talk about contraception, and they sorted it out for her. She said something like, ‘After five kids, I don't want any more.' That stuck with me. She came here and benefited from the services.”*

*MN, 20 years, Female, No children, Housewife*

“*There were unwanted pregnancies. People tried to find solutions on their own, tried to induce abortions. They would learn something from somewhere, use medicines or the morning-after pill. People were making attempts to end pregnancies on their own.”*

*OK, 48 years, Female, Physician*

“*I've had six abortions. I have two children. After that, I started using condoms as a method, with encouragement from friends. In short, there are so many things we don't know but need to know. These meetings shouldn't be missed. That's why I made sure to attend whenever I heard about them.”*

*GS, 31 years, Female, Two children, Housewife*

In addition, women were systematically provided with services included in The MISP. In this context, 4.7% of women received maternal and child health services, and 3.6% received hygiene kits.

“*…There were women about to give birth who couldn't find a hospital or couldn't get examined. I know many of my friends also benefited from here. For example, in the early days I didn't have the chance to shop. Yes, we had a little money, but there was no place to shop. Mothers with babies needed all sorts of things. Later on, I saw that you provided such support, like a bag prepared for babies. Many of my friends benefited from that too. It really touched me, and I will never forget it in my life.”*

*NK, 45 years, Female, Two children, Housewife*

“*The kits that were distributed were sufficient. They included everything from dental care items to razors, pads, underwear, soap, shampoo, and even a blanket.”*

*MB, 28 years, Female, One child, Housewife*

Women generally lacked awareness and knowledge regarding hygiene, while the high cost of sanitary products created additional barriers. The distribution of sanitary pads and hygiene materials was considered highly beneficial and contributed to improving hygiene practices.

“*I mean, generally, whether in this container or among the women I know around me, they don't really care about contraception or hygiene. I can say this clearly. They don't fully grasp what hygiene is, what it means, or what they need to do.”*

*SB, 40 years, Female, Three children, Hairdresser*

“*We try to get hygiene supplies to everyone in every possible way. Because even buying just one pack of pads is so expensive right now. If there are two teenage girls at home, that already makes three women. That means three packs. And that comes to about 150–200 TL (4–5 USD). The support provided here was really very helpful for them.”*

*ZG, 27 years, Female, Nurse*

“*For example, when NGOs distributed sanitary pads, when they gave us that kind of support, it was really amazing. We showed the girls how to use them properly in a hygienic way. Even though these things may seem small, in fact they mean a lot for the community.”*

*EA, 32 years, Female, Nurse*

Through mobile services, 83.0% of women received information on sexually transmitted infections, family planning, and early detection and screening programs for breast and cervical cancer.

“*For me, the breast examination and being informed about it was really good. Because a lump was found in my breast. I only noticed it after I came here. I didn't really know how to do the examination properly before.”*

*SB, 40 years, Female, Divorced, Three children*

“*We mostly have certain trainings. Generally, we give information about hand and genital hygiene, breast self-examination, Kegel exercises, and family planning counseling. If the age average is higher, we inform them about the menopausal transition. When we go to schools, we try to explain the menstrual cycle to young girls as much as possible.”*

*ZG, 27 years, Female, Nurse*

Another critical issue concerns women's sexual life and privacy. Living in single-room containers together with parents, children, and, in many cases, extended family members such as nieces, mothers-in-law, and fathers-in-law has significantly undermined both sexual life and privacy.

“*Being a woman here is really hard. I'll say this a bit embarrassed, but honestly there's no sex life at all. Because it's a one-room container, and there are kids. For example, I have two children, and they're just entering adolescence. On top of that, my husband's two nieces are with us… two teenage girls. We're six people in the same container. So of course, it's very difficult. You put aside your own feelings and emotions because you're focused on your children's needs, your husband's needs. And even when you try to be intimate, there's always the worry that a child might wake up. So really, there is no sexual life.”*

*NK, 45 years, Female, Two children, Housewife*

Serious safety concerns were identified. Frequent reports of harassment and abuse, particularly targeting women and young girls, were documented. In response, training and support were provided to women on this issue.

“*Before, there used to be guards, there was security. Now the police come from time to time, they patrol, but it's not as safe as it used to be. We already know that after a while we'll just be left to our fate… We can't leave our kids alone. An old man harassed a little girl. He was expelled, sent away from here. But I still can't leave my children alone, I can't even let them go to the park.”*

*SA, 19 years, Female, One child, Housewife*

Violence and harassment, in addition to safety concerns, were frequently reported as prevalent problems in temporary settlements.

“*A 15-year-old girl was kidnapped and abused for a week. It was a very specific case. Unfortunately, her family rejected her. Even if we wanted to do something, to help her, the process became even harder for her. That was something that really upset me.”*

*NE, 27 years, Female, Nurse*

“*My 14-year-old sister was forcibly taken away. We filed a complaint. Now she has been placed under the protection of Social Services by the state. The authorities said, ‘We have psychologists, we have doctors, they will see her.' After that, they moved her to another city.”*

*SA, 19 years, Female, One child, Housewife*

Finally, the emotions expressed by women regarding the service gap underscore the urgent importance of addressing needs in this area.

“*Just as much as we need support for women's health, we also need support psychologically, wherever we are lacking.”*

*GS, 31 years, Female, Two children, Housewife*

#### 3.2.2 Social determinants of health

The most significant factors negatively affecting access to SRH services were social determinants. Poverty emerged as the primary barrier to accessing SRH services. Women were forced to live in poor hygiene conditions due to poverty, and particularly undocumented and irregular migrant women faced major difficulties in accessing services because of lack of social security. Challenges in accessing clean water and hygiene facilities (such as bathing and laundry) in temporary settlements were identified as the main causes of vaginal discharge and infections.

The second most important social determinant affecting women's health was gender roles. Women were constrained by traditional gendered responsibilities such as childcare and household chores.

“*Yes, yes. It's always the same things. Wake up, breakfast, dinner, taking care of the child. Always the same. There's no activity at all.”*

*FA, 24 years, Female, One child, Housewife*

Important cultural barriers were encountered in conducting interviews with women during service delivery.

“*When we talked to a woman, we identified that she needed oral contraceptives, but after about a minute or two her father-in-law came over asking, ‘What are you doing? What are you here for?' Then he sent the woman straight inside, closed the door, and didn't say anything else to us, it was like he kicked us out. We do come across people with this kind of mindset. You could say there's this belief that a woman should just stay at home and do housework. Her husband or others like him actually don't allow her to access reproductive health services.”*

*AU, 30 years, Male, Physician*

In addition to poverty and lack of education, the language barrier constitutes a critical challenge for Syrian migrant women. Limited knowledge of the health system and challenges related to cultural adaptation were identified as significant barriers.

“*Right after the earthquake, the biggest problem we faced was disadvantaged groups accessing healthcare. Among these disadvantaged groups are women and migrants. The language barrier is another problem. If it's a woman and her language is Arabic, then two disadvantages overlap.”*

*AK, 58 years, Male, Physician*

Healthcare workers observed high fertility among Syrian women and noted limited prior awareness of modern contraceptive methods, such as oral contraceptives or the three-month injectable.

“*When you look at Syrian refugees, they feel good about having many children. For example, a 20–25-year-old woman might already have five or six children. For them, this is seen as a symbol of strength. The more children they have, the stronger they feel.”*

*MK, 26 years, Female, Midwife*

“*Among Syrians, we observe that they have an active sexual life — we always get this kind of feedback. But many of those women heard about the three-month contraceptive injection for the first time from us, and they started receiving it regularly, or they took oral contraceptive pills.”*

*ZG, 27 years, Female, Nurse*

Syrian women were perceived to experience higher levels of spousal pressure.

“*I know this because I've been around them a lot. I once invited a Syrian woman to a training session. At first, she said yes, but then she told me her husband wouldn't allow it. Why? I said, ‘We'll just be talking among women.' But normally when there's a meeting or something like that, husbands don't let them go. They say things like, ‘What are you doing there? What's this nonsense?' And it's not just about women's health meetings. For example, when a lawyer came to talk about domestic violence, they came several times, but husbands always put up barriers so that women don't become aware. They don't want them to improve themselves, to learn something, to hear something, or to know their rights.”*

*SB, 40, Woman, Hairdresser, 3 children*

Low health literacy constitutes a significant challenge; however, under such circumstances, educational interventions appear to be more effective.

“*Health literacy is low here, but there's something like this: one person tells another, then that person tells someone else, and before you know it, more people show up than expected. For example, maybe someone didn't come here for condoms or birth control pills, but when it's explained here, they realize, ‘Oh, this could actually be useful for me.' One of the things collective living brings is that they share this knowledge with each other. That's why I think education is really important.”*

*AY, 30, Female, Psychologist*

The inadequacy of housing conditions in container camp settlements was consistently emphasized.

“*We need cleaning supplies for the shared bathrooms and toilets. Without them, who's going to clean? We ended up washing the bathrooms ourselves. But five minutes later, they're dirty again. And we're spending money, too. Just washing with water doesn't really work. After the earthquake, we couldn't even find water… Our container is just one room. Eating, drinking—everything happens inside, and it makes us uncomfortable. My husband and child both has asthma. When I cook inside the container, it gets really hard. Because of the smell, we're constantly getting sick.”*

*ZSH, 49, Woman, 5 children, Housewife*

“*The bathrooms and toilets here are all shared. I mean, there's only one bathroom, and about 50–60 containers use the same one. That's why there are so many infectious diseases and infections here.”*

*EA, 32, Woman, Nurse*

#### 3.2.3 Community-based mobile services

The primary barrier to women's access to services was transportation difficulties. In crisis situations, it was extremely challenging for women with reproductive health problems to reach hospitals or family physicians. Therefore, the deployment of mobile health teams to circulate through camps significantly improved accessibility. In this intervention, two separate mobile teams visited container settlements and tent camps four working days per week, following a planned schedule. These visits were organized in coordination with the Provincial Health Directorate, local mukhtars, AFAD, and female community leaders within the camps.

“*I think the mobile service you provided, especially during that time, was really helpful, both in terms of giving information to the public and providing the materials needed for family planning.”*

*BS, 40, Female, Doctor*

“*The mukhtars really helped us with things like finding a place, gathering women, and making announcements. We also came up with this idea of a ‘leader woman' (female community leaders) or what we called a ‘smart woman.' These were women who worked as shopkeepers or were just very active, energetic, and well-connected in the neighborhood. We reached out to them and asked them to bring together their friends and groups.”*

*ZG, 27, Female, Nurse*

Procedures that required hospital settings, such as intrauterine device (IUD) insertions, were carried out by arranging appointments at hospitals and facilitating women's transfer. However, in cases where hospital transfers could not be organized, women were unable to access these services due to transportation difficulties and the absence of spousal assistance.

“*I had an IUD inserted at the hospital thanks to these services. The support was great—they took me there and brought me back, everything was fine. But there's one more thing I wish for. For example, I would have liked to go together again at least for the first follow-up check-up. At that time, my husband was very busy, so he couldn't take me. And back then there was also a problem with transportation—we didn't know which vehicle was going where.”*

*NK, 45 years old, Woman, Two children, Housewife*

Suggestions were made regarding the inclusion of a psychologist in the service team.

“*…For example, I came here and benefited from the women's health services, but there were times when I was really in psychological distress. I felt like I needed medication, or I realized I was having panic attacks. It would have been good if I could have gotten support for that too.”*

*NK, 45, Woman, 2 children, Housewife*

The need for psychosocial support for women, particularly in cases of domestic violence, was repeatedly expressed.

“*I mean, there are always arguments between husbands and wives. Here, it would help if women could openly talk about those arguments to someone and find a solution. It could be a kind of support that helps maintain peace and happiness in the family.”*

*MN, 20, Woman, Housewife*

The advantages of having an all-female service team were also highlighted.

“*When it's a woman in front of us, we can talk more comfortably, share more openly, and really open up. We can even go a bit deeper. That's why it felt easier, more comfortable.”*

*SB, 40, Woman, 3 Children, Hairdresser*

#### 3.2.4 Inter-sectoral collaboration

In the post-earthquake period, the destruction of both physical and organizational infrastructure in the public and private health sectors constituted a major barrier to service delivery. Consequently, the support of NGOs became crucial in maintaining healthcare provision in the region. SRH services initiated with the support of HASUDER, Direct Relief, UNFPA, and the Hatay Metropolitan Municipality were sustained for approximately two years, with extensions granted every six months through permits obtained from public authorities. Free supplies provided by international donor organizations were distributed to women in container settlements, ensuring access to essential services. This underscores the critical role of NGOs in sustaining healthcare delivery under crisis conditions.

“*But during my student years, in my research and internships, there wasn't even an ‘N' of NGO. I think it was really after the earthquake that we got to know NGOs this much and saw how effective they can be.”*

*AY, 30, Woman, Psychologist*

“*After we started working with NGOs, the number of applications increased a lot, and people's attitudes toward us completely changed. NGOs bring them such relief and such big benefits, both in terms of contraception and overall health. Right now, most women can't even afford to buy birth control pills… So having the service come right to them is extremely important.”*

*EA, 32, Woman, Nurse*

An important contribution of the services provided by NGOs was the fostering solidarity among women.

“*These women are wounded women… This place gave them a sense of friendship, a sense of responsibility, and also helped them learn a bit about working collectively. We have a committee made up of women. When we meet with institutions and organizations to identify the needs in this container city, we try to involve women in those discussions as well. We want women to strengthen their own leadership.”*

*SÇ, 26, Female, Social Worker*

It was recommended that non-governmental organizations (NGOs) enhance coordination with one another to improve service delivery.

“*For example, I came here with a women's health issue. If NGOs came together, when you explain your problem to one unit, they could refer you to another. They could say, ‘You can benefit from this side.' You know, there could also be more unity.”*

*NK, 45, Female, 2 children, Housewife*

The provision of free supplies alongside service delivery contributed to the greater effectiveness of the program.

“*When it comes to counseling whether it's pregnancy education, postpartum education, or family planning giving the training first and then providing one-on-one counseling, and finally handing over the materials, made things easier for both sides.”*

*AR, 28, Female, Nurse*

“*We give the training, but if you can't provide the necessary materials afterwards, it's difficult for people. They don't have access, so the education you've given stays a bit up in the air… For example, the doctor gave me the training, and then also provided the materials and support I needed. That's why it could be put into practice. We benefited a lot from this.”*

*EA, 32, Female, Nurse*

The support of international organizations for both service delivery and in-service training contributed to the overall success of the program.

“*While providing services, we kept building on what we had. So I think we didn't really have shortcomings, except for one thing—and that was LGBT issues. We had specifically asked for it, because it was something we realized in the field and felt we were lacking. We later completed those trainings through UNFPA.”*

*NE, 27, Female, Nurse*

A lack of adequate preparedness prior to the disaster was identified as a major concern.

“*In situations like these disasters, the state, the public sector, the private sector, and all civil society—basically all the groups working on reproductive health—should have had a plan and a program in place, so that these kinds of efforts could start as soon as possible after the disaster.”*

*AU, 30, Male, Physician*

The poor conditions experienced in the aftermath of the earthquake also had adverse effects on community mental health. Both the women interviewed and the healthcare personnel were found to be emotionally challenged.

“*We've been living like this for two years. I mean, we don't even look at ourselves in the mirror anymore. Things are so bad that we can't even bear to look.”*

*ZSH, 49, Female, 5 children, Housewife*

“*Being a woman is hard, and during a disaster it's even harder. Honestly, I can't cope. Sometimes a woman in front of me cries, and I hold back my tears. Then I turn around, go somewhere else, and cry on my own. Personally, I had to get psychological support.”*

Ö*Y, 36, Female, Nurse*

“*I think women have been carrying way too much. They went from living in their homes to suddenly living in tents or containers. Tiny spaces, stuck together with three to five kids. And almost every family lost someone close. They felt completely helpless. Some even wanted to restore their fertility like reopening their tubes because of losing a child.”*

*OK, 48, Female, Doctor*

Other quotes that reveal the themes of the research in more depth are presented in Table 2.

**Table 2:** Emergent themes and representative participant quotations reflecting experiences with sexual and reproductive health services in Hatay after the 2023 earthquakes.

**Theme**	**Quotation**
**The Gap in SRH services**	“*Neither our young people nor our married women know enough about contraception. Honestly, it's such a shame. … For example, my friend is 50 years old, she has two children, and she had two abortions. Why can't you protect yourself? Why aren't you aware of this? There are so many methods available.” SB, 40, Female, 3 children, Hairdresser*
		“*Condoms, pills, I mean, we couldn't get hold of these. We struggled to even find a pharmacy or the medicines. We literally felt like beggars. We couldn't access most things anyway. So when something came from you, it was really valuable for us.” VS, 28, Female, 2 children, Housewife*
		“*People have unprotected sex without thinking, and you know withdrawal isn't really a method. Then they come and say, ‘I got pregnant, can you remove the baby?' They use abortion as if it's a method of contraception.” CM, 58, Male, Physician*
**Social determinants**	“*I'm at home, taking care of my child. We wake up in the morning, go to school, I drop him off. … After that he comes back, and all my focus is on him.” SPK, 26, Female, 1 child, Housewife*
		“*Syrian women are either constantly pregnant or have just given birth. For them, a woman's duty is to reproduce. Some are 17 years old and already pregnant for the second time.” AY, 30, Female, Psychologist*
		“*Honestly, as women, the earthquake hit us really hard, but so did our health problems. It's like they weighed on us as heavily as the earthquake itself. Because you know, everything falls on women, home, children, husband, family, cooking. And after the earthquake, you don't even have a private life anymore.” GS, 31, Female, 2 children, Housewife*
**Community-based mobile services**	“*I think the most crucial thing in disasters is definitely mobile services. I really enjoy it when I go somewhere. You go with a doctor, you've got loads of supplies with you, and you meet so many women” ÖY, 36, Female, Nurse*
		“*When mobile teams were formed, they went to certain container cities or large settlements. If we could increase the number of mobile teams and visit more frequently, it would be much better.” AU, 30, Male, Physician*
		“*I wish there was at least a sexual therapist in the team, or a psychologist. Because when we came across special cases, like women with vaginismus, we had to refer them to other NGOs and couldn't do the follow-up ourselves. That's why I'd really like to have a psychologist or a sexual therapist with us. Also, the unit should be developed further to include adolescents, not just women.” ZG, 27, Female, Nurse*
**Inter-sectoral collaboration**	“*In every area, there's always a woman who stands out. We connect with them. When women hear that something is planned specifically for them, they say, ‘Are they really coming for us? Do they have something to tell us?' And that alone makes them happy. That's why NGO projects are so important. The feedback we got showed us this: ‘We attended these trainings, they explained things to us.' We loved hearing that. Our projects and your work complemented each other. There were so many positive sides to it. Physically providing access to oral contraceptives was so important, and you made that possible. That was really positive. Talking to women, reaching out to them, even just listening to them—it mattered a lot.” AK, 58, Male, Physician*
		“*For example, when we went out to provide mobile services, women in those places would say, ‘You came here before at such-and-such a time, we also want this service.' And we were so happy to hear that. It meant we were reaching people, expressing ourselves well, and that others could come and benefit from the same service too. That was really motivating for us.” AR, 28, Female, Nurse*
		“*Maybe we don't see this problem before a disaster, but afterwards it all comes to the surface.” CM, 58, Male, Doctor*

## 4 Discussion

The earthquake both exposed and exacerbated existing gaps in SRH services, underscoring the necessity of developing sustainable and inclusive emergency health responses. The findings highlight that social determinants—particularly gender, socioeconomic status and migration constitute critical factors influencing access to SRH services. Inter-sectoral collaboration and coordinated interventions proved essential in mitigating service disruptions and fostering community resilience during crisis situations. NGOs played a pivotal role in strengthening system capacity and facilitating service delivery in the aftermath of the disaster. The post-earthquake period, in particular, represents a crucial window for the deployment of community-based mobile health teams to ensure the continuity and accessibility of SRH services.

It was observed that 83.0% of service recipients benefited from mobile services, and interviewees consistently emphasized their importance. Multiple factors underscored the need for outreach, including damage to urban infrastructure and transport systems, limited availability of vehicles and roads, competing priorities in the aftermath of the earthquake, and the lack of financial resources to access health facilities. Even 2.5 years after the earthquake, the continued presence of 99 container settlements in the city center and 180 across Hatay Province illustrates the ongoing necessity of mobile health services. Evidence from other contexts, such as floods in Pakistan and India, similarly highlights the value of mobile service provision, government–NGO collaboration, and distribution of hygiene supplies in supporting women and girls (25).

Within this context, mobile services, with the principle of “reaching those who cannot come,” substantially increased service coverage. The integration of education with the provision of medicines, family planning methods, and hygiene supplies constituted a solution-oriented model. The identification of “lead women” (female community leaders) and engagement of these women to reach others fostered community participation in health services. This NGO-based service model is considered to have made a substantial contribution to public health in the post-earthquake period. Documenting both the quantitative outcomes and the qualitative insights of service users and providers adds value to the literature and offers an important model for the organization of health service delivery in future emergencies.

The fact that nearly all women resided in temporary shelters (containers or tents), combined with inadequate living conditions, limited access to water, bathing, and laundry facilities, as well as restricted access to cleaning supplies, sanitary pads, and underwear due to poor economic conditions, contributed to the high prevalence of infections.

Access to reproductive health services decreases in post-disaster settings, disproportionately affecting women with poor socioeconomic status (10). Other studies have also demonstrated an increase in STIs following earthquakes (26). For instance, a study conducted after the 2008 Wenchuan earthquake in China reported that the prevalence of lower genital tract infections (50.0% vs. 26.5%) and pelvic inflammatory disease (35.9% vs. 19.4%) nearly doubled. These infections tend to increase after disasters due to adverse living conditions, while women's lack of knowledge and awareness further contributes to their frequency (27). Similarly, a study conducted after the 2017 Kermanshah earthquake in Iran found that only 2% of affected women considered themselves at risk for STIs (9). Women diagnosed with STIs and UTIs in the post-earthquake period is 25%. Pre-earthquake infection rates in the region are similar to these rates (28, 29). Although there is no data specific to Hatay province, according to the latest Turkey Demographic and Health Survey data, which is conducted periodically in a representative sample of the country, Family Planning (FP) coverage is 70.0% and unmet FP need is 12.0% (22). These findings highlight the need for education and the importance of improving health literacy in this area. In HASUDER's service delivery model, all women receiving services were also provided with education on these issues.

These educational activities were observed to increase awareness, knowledge, and demand for services related to SRH. In addition to training, the distribution of family planning supplies and hygiene materials likely contributed to a reduction in infection rates and an increase in the use of family planning methods. Access to family planning services is a fundamental health right. However, in the aftermath of disasters, such access may be completely or partially disrupted. Limited availability of contraceptive methods leads to unintended pregnancies, induced abortions, and increased pregnancy-related health risks. Ensuring access to family planning services not only supports public health but also promotes gender equality, strengthens health systems, and contributes to economic development (7). An assessment by IPPF estimated that following the February 6 earthquakes, the unmet need for family planning in Hatay rose to approximately 50% (30).

Evidence from other disaster settings highlights similar challenges. Following the 2010 Haiti earthquake, access to the most widely used modern contraceptive methods, including injectables and condoms, was reduced, leading to increases in pregnancies, unintended pregnancies, and unmet family planning needs (8). Likewise, a study conducted after the 2017 Kermanshah earthquake in Iran reported that 14.4% of women experienced difficulties in accessing family planning methods (9). Further evidence from the 2012 earthquakes in Iran demonstrated a decline in contraceptive coverage (31).

Pregnancy testing was provided when needed, and a small number of women used emergency contraceptive pills following unprotected intercourse. Inadequate menstrual hygiene management (MHM) can result in adverse health outcomes such as skin irritation, urogenital infections including bacterial vaginosis and urinary tract infections, and reproductive complications (32, 33). To address these needs, 77.0% of women presenting to HASUDER service units were provided with cotton underwear and 22.0% with sanitary pads free of charge. As reported by women themselves, one of the key challenges in the post-disaster context was not only financial constraints but also the unavailability of products, as even online orders could not be delivered due to the suspension of courier services. Thus, these material supports were considered highly valuable. Similar findings have been reported elsewhere. In Iran, 34.1% of women were found to have limited access to sanitary pads following the 2017 Kermanshah earthquake (9). A study conducted in Bangladesh revealed that women affected by floods faced significant difficulties in accessing menstrual hygiene products, often resorting to unhygienic alternatives such as cloth or newspaper, thereby increasing health risks (25). These findings underscore the need for gender-sensitive disaster management policies that incorporate menstrual hygiene education, sustainable access to menstrual products, and the integration of MHM into reproductive health services within disaster response efforts (25, 34).

Emergency Birth Kits and Dignity Kits provided by UNFPA were distributed to pregnant and postpartum women. The Emergency Birth Kit includes soap, a sterile plastic sheet, a razor blade, an umbilical cord tie, a blanket, and latex gloves. Such kits are designed to save lives when women are compelled to give birth under challenging conditions, as they provide essential supplies to prevent infection—one of the leading causes of maternal mortality worldwide (35). UNFPA's Dignity Kits vary depending on community needs but generally include menstrual pads, bath soap, multiple pairs of underwear, detergent powder, sanitary napkins, a flashlight, toothpaste, a toothbrush, and a comb, packaged in a backpack or portable bucket. Each kit is tailored to meet the specific needs of women and girls (36). However, the limited number of kits available and the focus on pregnant and postpartum women meant that distribution coverage remained low compared to the number of women seeking support.

The fact that all staff members were proficient in Arabic effectively eliminated language barriers, particularly for Syrian migrant women receiving services. Moreover, the presence of an all-female health team facilitated communication on sensitive SRH issues, which constitute a private domain for women. In contrast, the involvement of male health personnel was found to negatively influence women's willingness to seek services or request hygiene supplies, especially in rural areas (34). A study conducted in Vanuatu similarly reported that women preferred MHM kits to be distributed by female staff (37).

Following the major earthquake of February 6, 2023, health services in Türkiye were severely disrupted. Hatay Province, one of the largest provinces with a pre-earthquake population of 1,686,043 in 2022 (38), had a significant population in need of SRH services. However, as expected, the scale of destruction led to SRH services being deprioritized, with public institutions initially focusing on the treatment of acute medical conditions. Preventive care, including SRH, could only be gradually restructured. In addition to the magnitude of the disaster, pre-existing structural weaknesses in the health system further contributed to this delay.

Public resources may be insufficient after major disasters, so reaching out to international funders has become especially crucial in the first months after an earthquake. However, it is essential to collaborate with all local administrative units and local health authorities. Mobile services and community participation must be ensured in the post-disaster period. Moving forward, SRH services should be transferred to the Public-government systems, ensuring free and inclusive, rights-based service delivery. This is particularly important for less developed countries like Nepal, Iran, Haiti, and Türkiye, where income distribution is more skewed and disadvantaged groups are at greater risk (8, 9, 12, 31). Therefore, it is even more crucial for governments to take responsibility.

Cooperation and solidarity efforts should be increased, particularly within the context of Office for the Coordination of Humanitarian Activities (OCHA) under the United Nations (39). Coordination among local resources should be ensured in such activities. Integration or alignment with national SRH or MISP clusters should be supplied for completeness.

### 4.1 Limitations

The quantitative data of the study could not be statistically analyzed in detail based on variables such as age and ethnic groups due to the insufficient number of variables in the databases of the institutions providing services in the region and their limited sharing of variables with the research team. Besides, due to the challenging living conditions and service delivery constraints in the post-disaster period, it was not possible to create and consistently use follow-up files for women who sought care. In addition, repeated visits could not be distinguished. Due to the extraordinary circumstances under which service delivery took place, a recording system that would allow this was not available. Due to the lack of internet access in most cases, records were kept manually. For the same reason, the first three months of quantitative data from the HASUDER project could not be included in this study due to missing records.

Furthermore, detailed SRH data from the pre-earthquake period were not collected. Therefore, a comparison of SRH status before and after the earthquake could not be conducted.

## Data Availability

The raw data supporting the conclusions of this article will be made available by the authors, without undue reservation.

## References

[1] AFAD. 06 Subat 2023 Kahramanmaraş (Pazarcik ve Elbistan) Depremleri Saha Çalişmalari Ön Degerlendirme Raporu. (2023). Available online at: https://deprem.afad.gov.tr/assets/pdf/Arazi_Onrapor_28022023_surum1_revize.pdf (Accessed June 10, 2025).

[2] UNDRR. Why Earthquakes Still Matter. (2025). Available online at: https://www.undrr.org/news/why-earthquakes-still-matter (Accessed August 17, 2025).

[3] T.C. Iç Işleri Bakanligi (2024). Available online at: https://www.icisleri.gov.tr/turkiyenin-birlik-ve-dayanisma-gucu-depremle-sinandi-asrin-felaketi-asrin-dayanismasina-donustu8 (Accessed August 2, 2025).

[4] BBC. (2024). Available online at: https://www.bbc.com/turkce/articles/c51rezpg45zo#:~:text=%22%C4%B0nsanlar%20%C3%B6fkeli%20ama%20yorguncan%20kayb%C4%B1%2024%20bine%20yak%C4%B1n (Accessed August 10, 2025).

[5] Association of Public Health Specialists (2025). Available online at: https://www.hasuder.org.tr

[6] Loewen S Pinchoff J Ngo TD Hindin MJ. The impact of natural disasters and epidemics on sexual and reproductive health in low- and middle- income countries: a narrative synthesis. Int J Gynecol Obstet. (2022) 157:11–8. doi: 10.1002/ijgo.13768 34043817

[7] Strid P Snead MC Galang RR. Fertility and contraception among women of reproductive age following a disaster: a scoping review. Reprod Health. (2022) 19:147. doi: 10.1186/s12978-022-01436-4 35739557 PMC9229126

[8] Behrman JA Weitzman A. Effects of the 2010 Haiti earthquake on women's reproductive health. Stud Fam Plann. (2016) 47:3–17. doi: 10.1111/j.1728-4465.2016.00045.x 27027990

[9] Rajabi E Hamidreza Khankeh HR Ranjbar M Mousavi M Norouzi M Farokhi . Evaluation of women's reproductive health status after the 2017 earthquake in Kermanshah, Iran. Health Emerg Disasters Q. (2022) 7:183–92. doi: 10.32598/hdq.7.4.271.2

[10] Warren E Post N Hossain M Blanchet K Roberts B. Systematic review of the evidence on the effectiveness of sexual and reproductive health interventions in humanitarian crises. BMJ Open. (2015) 5:e008226. doi: 10.1136/bmjopen-2015-008226 26685020 PMC4691726

[11] Swatzyna RJ Pillai VK. The effects of disaster on women's reproductive health in developing countries. Glob J Health Sci. (2013) 5:106–13. doi: 10.5539/gjhs.v5n4p106 23777727 PMC4776806

[12] Sohrabizadeh S Jahangiri K Khani Jazani R. Reproductive health in the recent disasters of Iran: a management perspective. BMC Public Health. (2018) 18:389. doi: 10.1186/s12889-018-5311-2 29562887 PMC5861605

[13] Ali M Bhatti MA Kuroiwa C. Challenges in access to and utilization of reproductive health care in Pakistan. J Ayub Med Coll Abbottabad. (2008) 20:3–7. Available online at: https://scispace.com/pdf/challenges-in-access-to-and-utilization-of-reproductive-patc5bfrhu.pdf 19999191

[14] Anwar J Mpofu E Matthews LR Shadoul AF Brock KE. Reproductive health and access to healthcare facilities: risk factors for depression and anxiety in women with an earthquake experience. BMC Public Health. (2011) 11:523. doi: 10.1186/1471-2458-11-523 21718519 PMC3146866

[15] Safajou F Nahidi F Ahmadi F. Reproductive health challenges during a flood: a qualitative study. Nurs Open. (2024) 11:e2044. doi: 10.1002/nop2.2044 38268287 PMC10697115

[16] Singh NS Aryasinghe S Smith J Khosla R Say L Blanchet K . A long way to go: a systematic review to assess the utilisation of sexual and reproductive health services during humanitarian crises. BMJ Glob Health. (2018) 3:e000682. doi: 10.1136/bmjgh-2017-000682 29736272 PMC5935157

[17] IPPF. MISP Readiness Assessment. Assessing Readiness to Provide the Minimum Initial Service Package (MISP) for Sexual and Reproductive Health in Emergencies. 2020 Version for Field Testing (2020). Available online at: https://www.fp2030.org/app/uploads/2023/07/MISP_readiness_assessment.pdf (Accessed June 17, 2025).

[18] Kalanlar B. Afetlerde Cinsel Saglik ve Üreme Sagligi. Turkiye Klinikleri J Obstet Womens Health Dis Nurs-Special Topics. (2018) 4:54–60. Available online at: https://www.turkiyeklinikleri.com/article/en-afetlerde-cinsel-saglik-ve-ureme-sagligi-81225.html (Accessed June 7, 2025).

[19] UNFPA. Reproductive Health for Communities in Crisis UNFPA Emergency Response (2025). ISBN 0-89714-619-0 E/10,000/2001. Available online at: https://www.unfpa.org/sites/default/files/pub-pdf/crisis_eng.pdf (Accessed June 1, 2025).

[20] IAGW. Expanding Access to Sexual and Reproductive Health in Crises. (2022). Available online at: https://iawg.net/ (Accessed May 10, 2025).

[21] UNFPA. Minimum Initial Service Package (MISP) for Reproductive Health in Crisis Situations. (2025). Available online at: https://www.unfpa.org/sites/default/files/resource-pdf/MISP-Reference-English.pdf (Accessed June 3, 2025).

[22] Hacettepe University. Turkey Demographic and Health Survey 2018. (2019). Available online at: https://fs.hacettepe.edu.tr/hips/dosyalar/Ara%C5%9Ft%C4%B1rmalar%20-%20raporlar/2018%20TNSA/TDHS2018_mainReport_compressed.pdf#page=101.12 (Accessed July 13, 2025).

[23] Sphere. The Sphere Handbook 2018. (2018). Available online at: https://spherestandards.org/handbook/ (Accessed July 5, 2025). doi: 10.3362/9781908176707

[24] WHO. HRP at 50: Sexual and Reproductive Health and Rights in Epidemic and Pandemic Preparedness and Response. (2023). Available online at: https://www.who.int/publications/i/item/9789240075122 (Accessed July 8, 2025).

[25] Al-Mamun M Kalam A Karim MZ Alam M Khan TH. Menstrual hygiene management in flood-affected Bangladesh: addressing socio-cultural barriers, infrastructure gaps, and policy responses. Front Public Health. (2025) 13:1538447. doi: 10.3389/fpubh.2025.1538447 40255381 PMC12006079

[26] Stephens JH Lassa JA. Sexual and reproductive health during disasters: a scoping review of the evidence. Int J Disaster Risk Reduct. (2020) 50:101733. doi: 10.1016/j.ijdrr.2020.101733

[27] Liu S Han J Xiao D Ma C Chen B. A report on the reproductive health of women after the massive 2008 Wenchuan earthquake. Int J Gynaecol Obstet. (2010) 108:161–4. doi: 10.1016/j.ijgo.2009.08.030 19892335

[28] Ege E Timur S Zincir H Egri M Sunar Reeder B. Women's douching practices and related attitudes in eastern Turkey. J Obstet Gynaecol Res. (2007) 33:353–9. doi: 10.1111/j.1447-0756.2007.00535.x 17578366

[29] Hadimli A Akan A Kardes G Akkurt B Saydam BK. Determination of menstrual hygiene management and genital hygiene behaviors of students: a university example from Turkey. Rev Esc Enferm USP. (2024) 58:e20240113. doi: 10.1590/1980-220x-reeusp-2024-0113en 39621939 PMC11611317

[30] IPFF. Unmet Need for Contraceptives Quadruples in Parts of Earthquake-Affected Türkiye (2023). Available online at: https://www.ippf.org/media-center/unmet-need-contraceptives-quadruples-parts-earthquake-affected-turkiye (Accessed July 25, 2025).

[31] Bahmanjanbeh F Kohan S Yarmohammadian MH Haghshenas A. Evaluation of reproductive health indicators in women affected by East Azerbaijan earthquake on August 2012. Iran J Nurs Midwifery Res. (2016) 21:504–9. doi: 10.4103/1735-9066.193414 27904635 PMC5114796

[32] van Eijk AM Sivakami M Thakkar MB Bauman A Laserson KF Coates S . Menstrual hygiene management among adolescent girls in India: a systematic review and meta-analysis. BMJ Open. (2016) 6:e010290. doi: 10.1136/bmjopen-2015-010290 26936906 PMC4785312

[33] Das P Baker KK Dutta A Swain T Sahoo S Das BS . Menstrual hygiene practices, WASH access and the risk of urogenital infection in women from Odisha, India. PLoS ONE. (2015) 10:e0130777. doi: 10.1371/journal.pone.0130777 26125184 PMC4488331

[34] Tufail Z Ahmer W Gulzar S Hasanain M Shah HH. Menstrual hygiene management in flood-affected Pakistan: addressing challenges and ensuring women's health and dignity. Front Glob Women's Health. (2023) 4:1238526. doi: 10.3389/fgwh.2023.1238526 37600522 PMC10433887

[35] UNFPA. (2025). Available online at: https://www.unfpa.org/donate/EmergencyBirthKit/a (Accessed July 21, 2025).

[36] UNFPA. What is in a UNFPA Dignity Kit? (2025). Available online at: https://www.usaforunfpa.org/whats-in-a-unfpa-dignity-kit/ (Accessed July 21, 2025).

[37] Downing SG Benjimen S Natoli L Bell V. Menstrual hygiene management in disasters: the concerns, needs, and preferences of women and girls in Vanuatu. Waterlines. (2021) 40:144–59. doi: 10.3362/1756-3488.21-00002

[38] Adrese Dayali Nüfus Kayit Sistemi Sonuçlari. (2022). Available online at: https://data.tuik.gov.tr/Bulten/Index?p=49685#:~:text=T%C3%BCrkiye'de%20ikamet%20eden%20n%C3%BCfus,575%20bin%20441%20ki%C5%9Fi%20oldu

[39] OCHA. OCHA's Strategic Plan 2023-2026: Transforming Humanitarian Coordination. (2023). Available online at: https://www.unocha.org/publications/report/world/ochas-strategic-plan-2023-2026-transforming-humanitarian-coordination (Accessed July 11, 2025).

