# Supplementary material for: Continuity of care in crisis: Community based mobile health teams for sexual and reproductive health services in post-earthquake period in Türkiye
Source: Front Public Health. 2026 Jan 15;13:1706038. doi: 10.3389/fpubh.2025.1706038 (PMC12852466; doi:10.3389/fpubh.2025.1706038)
Supplement: Supplementary file 1 [file Table_1.docx]

Supplementary File 1: COREQ checklist

Consolidated criteria for reporting qualitative studies (COREQ): 32-item checklist

| **Item No** | | **Guide Questions/Description** | **Reported on Page #** |  |  |
| --- | --- | --- | --- | --- | --- |
| **Domain 1: Research team and reflexivity** | | | |  |  |
| **Personal Characteristics** | | | |  |  |
| 1. Interviewer/ facilitator | | Which author/s conducted the interview or focus group? |  | All of the eight authors conducted interviews (DÇ, ACB, TI, NS, OT, BNB, ZK, BK) |  |
| 2. Credentials | | What were the researcher’s credentials? E.g., PhD, MD |  | All of the authors are Medical Doctors, 7 people are specialist in public health, one has PhD in Public Health. |  |
| 3. Occupation | | What was their occupation at the time of the study? |  | Seven of them have been working at the Department of Public Health as academic staff in different Universities. One has been working at the Department of History of Medicine and Ethics |  |
| 4. Gender | | Was the researcher male or female? |  | Five female and three male |  |
| 5. Experience and training | | What experience or training did the researcher have? |  | Five of the eight authors (BK, ACB, OT, TI, NS) have experience in qualitative research area nearly for 10 years. They published many qualitative papers in the indexed international journals. The others are young researchers. |  |
| **Relationship with participants** | | | |  |  |
| 6. Relationship established | | Was a relationship established prior to study commencement? |  | No, there is no relationship except HASUDER team |  |
| 7. Participant knowledge of the interviewer | | What did the participants know about the researcher? e.g. personal goals, reasons for doing the research? |  | Only HASUDER team know some of the researchers as professionally and their goals for doing the research. |  |
| 8. Interviewer characteristics | | What characteristics were reported about the interviewer/facilitator? e.g. Bias, assumptions, reasons and interests in the research topic |  | No |  |
| **Domain 2: study design** | | |  |  |  |
| **Theoretical framework** | | |  |  |  |
| 9. Methodological orientation and Theory | What methodological orientation was stated to underpin the study? e.g. grounded theory, discourse analysis, ethnography, phenomenology, content analysis |  | Phenomenological design |  |  |
| **Participant selection** | | |  |  |  |
| 10. Sampling | How were participants selected? e.g., purposive, convenience, consecutive, snowball |  | Purposeful and snowball sampling |  |  |
| 11. Method of approach | How were participants approached? e.g., face-to-face, telephone, mail, email |  | Face to face |  |  |
| 12. Sample size | How many participants were in the study? |  | 13 in-depth interviews, 5 focus groups, totally 34 participants. |  |  |
| 13. Non-participation Setting | How many people refused to participate or dropped out? Reasons? |  | No refuse |  |  |
| 14. Setting of data collection | Where was the data collected? e.g., home, clinic, workplace |  | In the temporary living areas, hospital buildings, university building, HASUDER service units |  |  |
| 15. Presence of nonparticipants | Was anyone else present besides the participants and researchers? |  | No |  |  |
| 16. Description of sample | What are the important characteristics of the sample? e.g. demographic data, date |  | A total of 15,841 women were recorded in the cross-sectional phase. The mean age of the women was 38.0 ± 12.9 years; 80.2% were Turkish citizens and 14.7% were Syrian migrants. In the qualitative phase, 5 focus group discussions involving health workers (one group, 5 people), Turkish women (3 groups, 12 people), and Syrian migrant women (one group, 4 people) were conducted. In addition, data were collected through 13 in-depth interviews (4 academics, 6 NGO, 3 service providers). Totally 34 participants were interviewed in the qualitative phase. |  |  |
| **Data collection** | | |  |  | No |
| 17. Interview guide | Were questions, prompts, and guides provided by the authors? Was it pilot tested? |  | Yes, there was a pilot test. |  |  |
| 18. Repeat interviews | Were repeat interviews carried out? If yes, how many? |  | No |  |  |
| 19. Audio/visual recording | Did the research use audio or visual recording to collect the data? |  | Yes, only audio recording |  |  |
| 20. Field notes | Were field notes made during and/or after the interview or focus group? |  | Yes, there was. |  |  |
| 21. Duration | What was the duration of the interviews or focus group? |  | A total of five focus group discussions were conducted, with an average duration of 77 minutes, and 13 in-depth interviews with an average duration of 52 minutes |  |  |
| 22. Data saturation | Was data saturation discussed? |  | Yes |  |  |
| 23. Transcripts returned | Were transcripts returned to participants for comment and/or correction? |  | Yes |  |  |
| **Domain 3: analysis and findings** | | |  |  |  |
| **Data analysis** | | |  |  |  |
| 24. Number of data coders | How many data coders coded the data? |  | Each interview was coded by at least 2 researchers and totally 8 of eight researchers coded the data |  |  |
| 25. Description of the coding tree | Did the authors provide a description of the coding tree? |  | Yes |  |  |
| 26. Derivation of themes | Were themes identified in advance or derived from the data? |  | Yes |  |  |
| 27. Software | What software, if applicable, was used to manage the data? |  | No. The data were coded manually and analyzed by thematic content analysis method. |  |  |
| 28. Participant checking | Did participants provide feedback on the findings? |  | Participants were confirmed the transcriptions but not for the findings |  |  |
| **Reporting** | | |  |  |  |
| 29. Quotations presented | Were participant quotations presented to illustrate the themes/findings? Was each quotation identified? e.g., participant number |  | Yes |  |  |
| 30. Data and findings consistent | Was there consistency between the data presented and the findings? |  | Yes |  |  |
| 31. Clarity of major themes | Were major themes clearly presented in the findings? |  | Yes |  |  |
| 32. Clarity of minor themes | Is there a description of diverse cases or a discussion of minor themes? |  | No |  |  |
